# Commentary: Results of Onlay Preputial Flap Urethroplasty for the Single-Stage Repair of Mid-and Proximal Hypospadias

**DOI:** 10.3389/fped.2018.00129

**Published:** 2018-05-14

**Authors:** Smail Acimi

**Affiliations:** Visceral Surgery, Children's Hospital Canastel, University of Oran, Oran, Algeria

**Keywords:** curvature, hypospadias, preputial flap, one-stage repair, onlay island flap urethroplasty

Our compliments to the authors for their great paper. A written manuscript by two leading specialists of surgical repair of external genitalia (1) with excellent photos which trace all the steps of onlay island flap urethroplasty and give value to the paper.

The proximal forms of hypospadias represent approximately a third of cases of this urogenital malformation. These forms are very often associated with curvatures (2). Thus, the treatment of proximal hypospadias includes two surgical stages: correction of the curvature and urethroplasty. If there is no consensus on the best technique for repair of proximal hypospadias, the choice of the surgical technique of urethroplasty depends on the method of curvature correction.

The essential factor responsible for the curvature associated with proximal hypospadias is the fibrosis tissue present on the ventral side of the penis. However, when the initial curvature is more than 90 degrees, a short urethral plate becomes the main cause of this curvature (2). Thus, there are cases where it was difficult to preserve the urethral plate in the curvature correction, especially when the initial curvature is >90° (2).

In 1987, Elder et al. (3) proposed the use of onlay island flap in the repair of mid and distal penile hypospadias without curvature. This idea had been resumed 4 years later by Mollard et al. (4) who applied this concept to the repair of hypospadias with severe curvature on a preserved urethral plate. In the early 1990's, the use of the onlay island flap in repair of the proximal hypospadias has experienced an important worldwide success.

For more than 20 years we have used the onlay island flap urethroplasty on a large number of patients and the first assessment of the results of our work was published in 2005 (5). We have never used the technique described by González et al (6), but the use of the technique described by Elder and Mollard gave us a complete satisfaction with a very low rate of fistulas, without proximal and distal stenosis. And the duration of the urinary diversion by a stent (6F or 8F feeding tube) which was at the beginning of 7 days has been reduced to 2 days. However, the glans dehiscence was more frequent than the rate reported by the authors. The better results obtained with the onlay island flap compared to tubulization of the prepuce are due to the ability to resect the poorly vascularized edges, because there is often an excess of the prepuce, without the risk of proximal stricture, and the presence of the solid floor made from the vestigial dysgenetic tissue from aplasia of the corpus spongiosum, termed the urethral plate (2, 5). Our attitude was: when the correction of the curvature by the release of the skin and dartos fascia was insufficient, the urethral plate was lifted up from the corpora cavernosa by fine scissors slipped between the urethral plate and corpus cavernous, and all the fibrous tissue would be resected (a gesture which gives a very low correction of the curvature of the penis, ranging from 0 to 20 degrees). However, this procedure was associated with dorsal plication by excision of a diamond shape at the point of the maximum bend dorsally (after complete separation of the dorsal neurovascular bundle from the corpus cavernosum) if an artificial saline test demonstrated a persistent penile curvature. A procedure of dorsal plication which seems more physiologic than others, allows preserving the urethral plaque, even with significant curvatures. However, it remains a difficult gesture which requires experienced hands and a significant number of adults and adolescents that we continue to follow. Patients who, at their young age, underwent onlay island flap urethroplasty with or without mobilization of the urethral plate and dorsal plication, report the presence of curvature during erection (Figure 1). I think that the reappearance of the curvature after a few years is due to poor growth of the urethral plate preserved. Despite that, those curvatures are not significant, and no patient has requested a surgical correction of the curvature. Our attitude in front of proximal hypospadias associated with curvature has totally changed in the last years. The correction of the curvature should be a priority and we do not hesitate to transect the urethral plate if the release of the curvature requires it. Thus, we do not practice the dorsal plication anymore. When the preservation of urethral plate is possible, this plate can be used as a solid floor for urethroplasty by onlay island flap which remains an excellent technique. It also can be tubularized. However, when the urethral plate was resected, the urethroplasty can be achieved by tubularized island flap or in two stages or by Koyanagi-Hayashi's procedure, a technique which seems to give good results, but we will need a large number of patients and a lot of time to make a real scientific assessment of the results of this technique.

**Figure 1:**
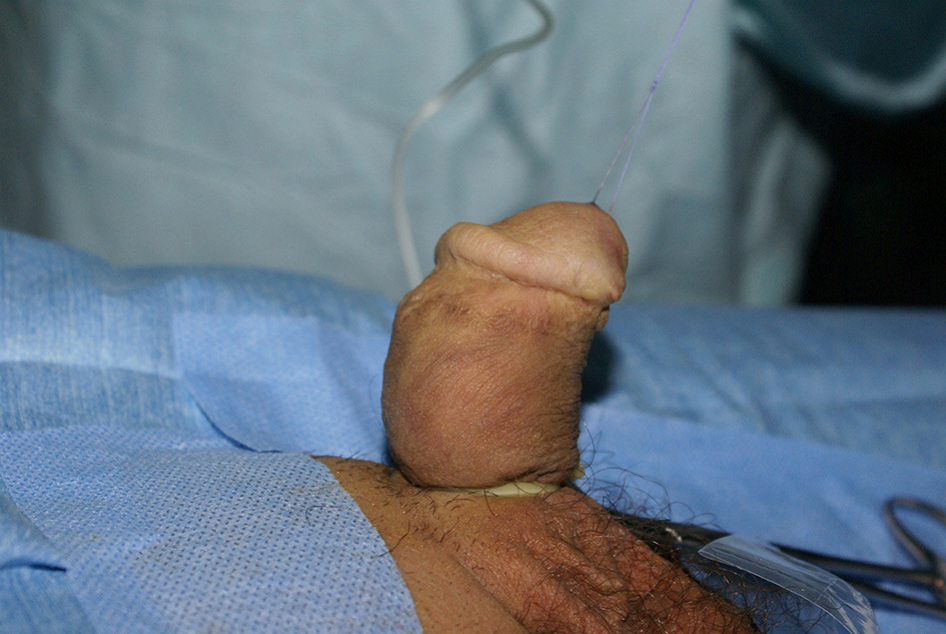
Artificial erection test in a 16-years-old adolescent which shows a reappearance of the curvature at the adolescence. This patient underwent a surgical correction at 1-year-old for proximal hypospadias associated with severe curvature (97 degrees) which was completely corrected by dorsal plication.

## Author contributions

The author confirms being the sole contributor of this work and approved it for publication.

### Conflict of interest statement

The author declares that the research was conducted in the absence of any commercial or financial relationships that could be construed as a potential conflict of interest.

## References

[1] González R Lingnau A Ludwikowski BM. Results of Onlay Preputial Flap Urethroplasty for the Single-Stage Repair of Mid-and proximal hypospadias. Front Pediatr. (2018) 6:19. 10.3389/fped.2018.00019 29473028 PMC5809423

[2] Acimi S Acimi MA. Can we preserve the urethral plate in proximal hypospadias repair? Ann Plast Surg (2017) 79:68–72. 10.1097/SAP.0000000000001055 28328642

[3] Elder JS Duckett JW Snyder HM. Onlay island flap in the repair of mid and distal penile hypospadias without chordée. J Urol. (1987) 138:376–9. 3599258 10.1016/s0022-5347(17)43152-1

[4] Mollard P Mouriquand P Felfelat T. Application of the onlay island flap urethroplasty to penile hypospadias with severe chordée. Br J Urol. (1991) 68:317–9. 1913075 10.1111/j.1464-410x.1991.tb15331.x

[5] Acimi S Boukli-Hacene A. Interest of mobilization of the urethral plate in the release of chordee related to posterior hypospadias. Prog Urol. (2005) 15:59–62. 15822394

[6] Gonzalez R Smith C Denes E. Double onlay preputial flap for proximal hypospadias repair. J Urol. (1996) 156:832–4. 10.1016/S0022-5347(01)65831-2 8683795

